# The Effective Depth of Skid Resistance (EDSR): A Novel Approach to Detecting Skid Resistance in Asphalt Pavements

**DOI:** 10.3390/ma18061204

**Published:** 2025-03-07

**Authors:** Yi Luo, Yongli Xu, Yiming Li, Liming Wang, Hongguang Wang

**Affiliations:** 1 College of Civil Engineering and Transportation, Northeast Forestry University, Harbin 150040, China; 2 College of Aerospace and Civil Engineering, Harbin Engineering University, Harbin 150001, China

**Keywords:** asphalt pavement, skid resistance, Effective Depth of Skid Resistance (EDSR), laser scanning, surface texture, fractal dimensions

## Abstract

Asphalt pavement skid resistance, governed by surface texture, is critical for traffic safety. Most research has focused on full-depth textural characteristics, often overlooking the depth of tire–pavement contact under real traffic conditions. This study introduces the concept of the Effective Depth of Skid Resistance (EDSR) to describe the effective depth of tire–asphalt contact, improving skid resistance assessment accuracy. Using blue linear laser scanning, surface textures of three common asphalt pavements with wearing courses—AC-13, AC-16, and SMA-13—were analyzed, and friction coefficients were measured using a British pendulum. After pre-processing three-dimensional texture data, fractal dimensions at various depths were calculated using the box-counting method and correlated with the friction coefficients. Previous studies show an insignificant correlation between full-depth asphalt pavement textures and skid resistance. However, this study found a significant positive correlation between skid resistance and pavement textures at specific depths or the EDSR. A depth with a correlation exceeding 0.9 was defined as the EDSR. Linear formulas were established for each pavement type within these EDSR ranges. A theoretical model was developed for predicting skid resistance, showing an over 80% accuracy against real-world data, indicating its potential for improving road surface performance detection.

## 1. Introduction

As one of the key road performance indicators of asphalt road, skid resistance has the most direct impact on driving safety [1,2,3]. The skid resistance of asphalt mainly depends on the texture structure formed by the asphalt mixture on the road surface, which is divided into macro-texture and micro-texture [4,5,6,7]. Macro-texture is the texture structure of the pavement from 0.5 mm–50 mm in the wavelength direction and from 0.2 mm–10 mm in the height direction [8]. It is mainly affected by asphalt mix gradation, mineral spacing, mineral distribution, and mineral shape and has a greater impact on the skid resistance of pavements under wet, high-speed conditions [9,10]. Micro-textures are pavement textures that are less than 0.5 mm in the wavelength direction and 0.005–0.2 mm in the height direction. They are mainly influenced by the surface texture of the mineral material, which has a greater impact on the skid resistance of pavements in dry, low-speed conditions [11,12]. Properly selected macro- and micro-textures are capable of enhancing the properties of pavements, offering enhanced skid resistance and a decrease in noise levels [13,14,15,16]. Large textures and unevenness, when present, can trigger irregular vibrations in the rubber tires and cause jolts within the vehicle. These phenomena have the potential to result in significant degradation in many of the aforementioned areas and should be evaded to the greatest extent possible [4,17,18].

The skid resistance of a pavement can be measured directly using skid resistance data, or it can be assessed indirectly through pavement characteristics. Zhang et al. [19] presented a 3D dense point scanning method and a novel metric for assessing the skid resistance of asphalt pavements. Their study delved into the sources of measurement errors associated with laser scanning equipment and proposed enhancements, including the implementation of median filtering to refine the data. Within the scope of their research, they conducted measurements on aggregates exhibiting four distinct levels of wear (labeled A, B, C, and D) and derived parameters like fractal dimension and peak angle to assess skid resistance. Subsequent measurements were taken on surfaces that had been subjected to varying degrees of wear, with calculations made for parameters such as the fractal dimension and average depth. After obtaining the 3D images, the pressure distribution on the surface of the track slab was measured using a pressure-sensitive film loaded on the pavement at different gradients. The findings indicate that the processes of abrasion and kneading result in diminished values for the fractal dimension, average depth, peak quantity, and friction coefficient of the aggregate and track plates. However, their research did not relate the friction coefficients to their calculated aggregate parameter values. Chen Bo et al. [20] utilized a pressure-sensitive membrane to measure the contact pressure at the interface between a radial tire and asphalt pavement, thereby determining the actual contact area and the distribution of stress. The fractal dimensions of the fracture surfaces of four types of roadway panels were estimated using an improved projection coverage method to characterize their surface roughness. The research findings suggest that the surface’s fractal dimension is a more effective measure of the texture granularity for both macro- and micro-textures, outperforming the traditional gravel method for evaluating pavement texture depth. However, their research did not compute the fractal dimension for real asphalt pavement textures. Zong et al. [21] conducted experiments to explore the mechanical characteristics and surface features of coarse aggregate types, including limestone, basalt, granite, and 88# calcined corundum. They utilized image processing software to determine the macroscopic morphological characteristic parameters (MACPs) of these aggregates, further examining their distribution patterns and traits. Through extensive polishing tests, the researchers quantitatively illustrated the dynamic evolution of both macro- and micro-morphological attributes of the coarse aggregates in relation to the polished stone value (PSV). Furthermore, they discovered a strong correlation, with a gray entropy correlation coefficient of 0.9037, between the PSV and the fractal dimension as the PSV declines. The study’s outcomes indicate that the fractal dimension can serve as a primary metric for assessing the long-term polishing resistance of coarse aggregates. This offers a valuable reference for predicting the enduring skid resistance of sustainable asphalt pavements.

From the results of the above studies, it is very feasible to characterize the roughness of asphalt pavement using the fractal dimension. The fractal dimension is an effective tool that can characterize complex systems, and it can be used to characterize the geometry and texture of asphalt pavements to better reflect their roughness.

Gao et al. [22] used a grinding machine to test the texture of 31 Texas asphalt pavements at various depths, drum types, and speeds, measuring macroscopic texture and friction before and at 3, 6, 12, and 18 months post-grinding. It was found that the sections after grinding with a fine roller exhibited higher performance in terms of post-grinding frictional resistance and macro-texture. The research presents the effect of texture parameters on the skid resistance of asphalt pavements at different depths of cut. Hainian Wang et al. [23] utilized advanced imaging techniques, namely the second-generation polymer imaging measurement system (AIMS II) and X-ray computed tomography (CT), to assess the shape of particles and track their morphological changes. They employed Analysis of Variance (ANOVA) to evaluate and compare the performance of these two imaging analysis systems. This approach provided a comprehensive analysis of the particle morphology, which is crucial for understanding the skid resistance parameters of asphalt mixtures. Complementing these advanced analyses, traditional gravel tests and British pendulum tests were applied to the polymers to discern the link between particle morphology and the skid resistance of asphalt pavements. The study revealed that while regression analysis could correlate the skid resistance of asphalt pavements with morphological properties, it overlooked the variable depths of tire–pavement contact encountered in real-world traffic conditions.

**Figure 1:**
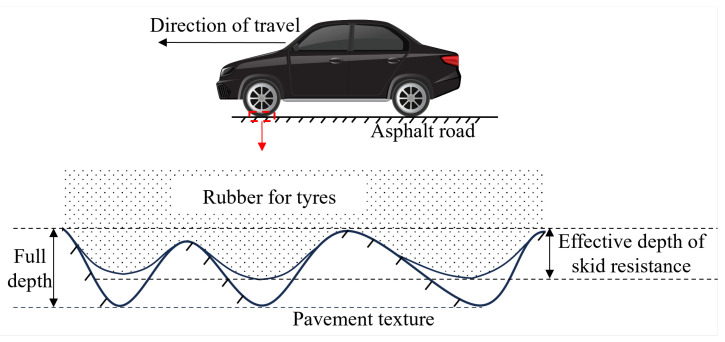
Schematic of EDSR (Effective Depth of Skid Resistance).

The above research shows that it is feasible to utilize the three-dimensional topographic parameters of asphalt pavements for the evaluation of their skid resistance and that the fractal dimension of asphalt pavements can represent their roughness well. Current research on the skid resistance performance of asphalt pavements has primarily focused on the texture characteristics across the full depth, neglecting the actual depth of tire–pavement contact under traffic conditions. Consequently, this study introduces the concept of the EDSR (Effective Depth of Skid Resistance) to quantitatively describe the effective depth of contact between tires and asphalt pavement under real traffic conditions, thereby enabling a more accurate assessment of the pavement’s skid resistance performance. A schematic representation of the EDSR is presented in Figure 1.

## 2. Materials and Methods

### 2.1. Framework

**Figure 2:**
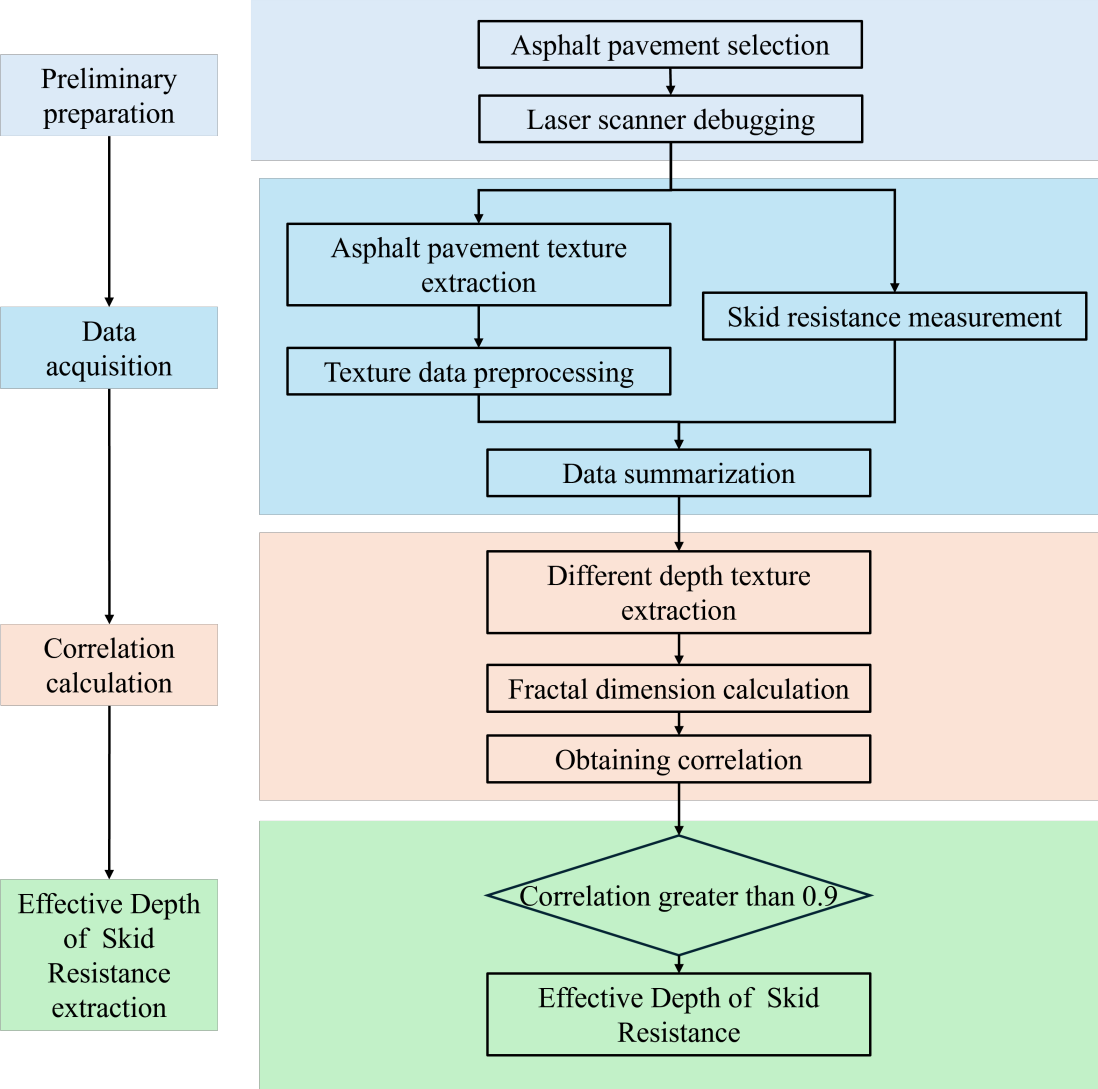
Effective Depth of Skid Resistance (EDSR) flowchart.

In this research, from this perspective, a laser texture scanner was utilized to extract 3D cloud maps of asphalt pavement, and 3D Gaussian filtering with least squares was applied to the initially extracted 3D cloud maps to eliminate noise and skew. The fractal dimension of asphalt pavement was calculated accurately using a generalization of the box-counting method. The 3D cloud maps were divided at 0.1 mm intervals to calculate the fractal dimension of the asphalt pavement at different depths. Elevation image analysis was used to find the causes of outliers in the process of finding the fractal dimension, and the outliers were eliminated. Finally, the correlation between the fractal dimension of the pavement texture and the skid resistance value of the pavement at different depths was analyzed. The outcomes of the calculations are expected to enhance the precision of forthcoming studies focused on the skid resistance of asphalt pavements. A depth range with a correlation coefficient greater than 0.9 was designated as the Effective Depth of Skid Resistance (EDSR). The computational process for determining the EDSR is illustrated in Figure 2.

### 2.2. Field Pavement Selection

To ensure the reliability of our results, we aimed to reduce error due to single sampling, stabilize the algorithm proposed in this paper, and ensure the universality of the indicators. Considering the significant differences in the void volume distribution of different graded asphalt mixtures, three types of asphalt roads, AC-13, AC-16, and SMA-13, were selected for this experiment. Three different mileage segments on each asphalt road were selected and 10 regions within each mileage range were selected for texture extraction. The three asphalt pavement gradations are shown in Table 1.

### 2.3. Pavement Texture Acquisition

Linear laser scanning, a non-contact metrological technique, facilitates the acquisition of three-dimensional topographical data by projecting a laser beam onto a target surface and subsequently capturing the reflected pattern. This methodology is of paramount importance in the realm of asphalt pavement texture analysis, providing detailed surface texture information that is vital for evaluating the skid resistance and overall integrity of pavements [24,25].

In the scanning procedure, a linear laser device emits a laser beam that, upon striking the pavement, manifests as a luminous linear projection. The deformation of this projection, attributable to the pavement’s topographical variations, is recorded by an array of cameras. Utilizing the principles of optical triangulation, in tandem with predefined geometric parameters relating to the camera and laser emitter, the elevation of each point on the pavement surface is computationally determined. This approach ensures a high degree of precision, which is indispensable for thorough pavement assessment and analysis [2,25,26].

**Figure 3:**
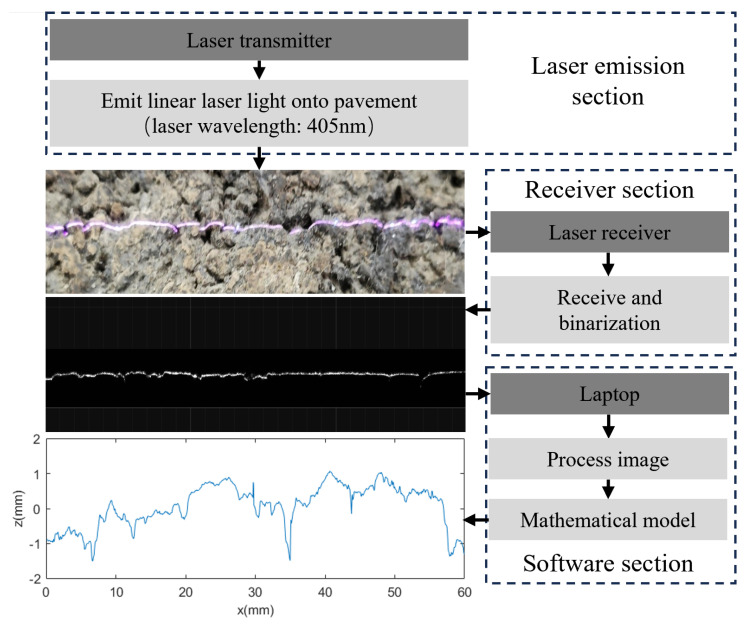
Schematic diagram of cross−section elevation data acquisition.

Figure 3 illustrates the procedure for acquiring cross-sectional elevation data of asphalt pavement using a linear laser scanner.

The mathematical model in Figure 3 is shown in Equations (1)–(5).

(1) $$K=\left[\begin{array}{ccc} f_{x} & 0 & c_{x}\\ 0 & f_{y} & c_{y}\\ 0 & 0 & 1 \end{array}\right]$$

fx and fy are the focal lengths along the width and height of the image. cx and cy are the principal point coordinates.

(2) $$x=\frac{u-c_{x}}{f_{x}}$$

(3) $$x=\frac{v-c_{y}}{f_{y}}$$

The image coordinates (u, v) are first converted from pixel coordinates to normalized image coordinates.

(4) $$Z=\frac{f_{x}}{x}$$

Z is the depth along the optical axis of the camera. X denotes a point in 3D space within the global coordinate system.

(5) H=Zcosθ

θ represents the angular deviation between the laser line and the optical axis of the camera.

**Figure 4:**
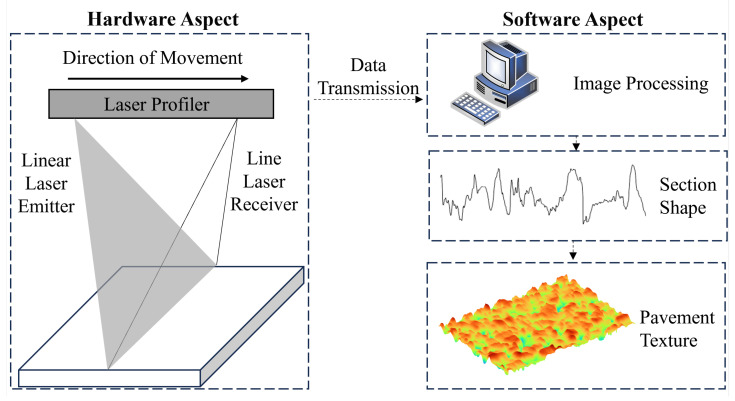
Schematic diagram of laser scanning.

After, the elevation of the corresponding cross-section of the line laser can be obtained from the picture of the line laser through the calculation of Equations (1)–(5). The asphalt pavement cross-section is extracted at intervals of 0.05 mm and spliced to obtain the complete asphalt pavement texture data. Figure 4 shows the schematic of the scanning process.

**Figure 5:**
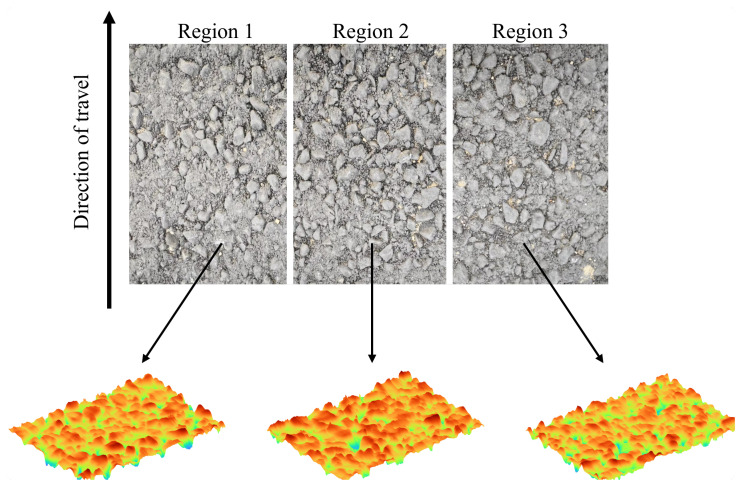
Scanning schematic of asphalt specimen.

For this experiment, three neighboring areas were selected for scanning and 30 such areas were selected on each road. As the maximum scanning range of the laser scanner was 104 mm × 72 mm, to ensure the integrity of the data that were output, the data range was defined as 100 mm × 70 mm. Figure 5 displays the scanning schematic diagram.

During the extraction of asphalt pavement texture, a pendulum friction meter was utilized to measure the British Pendulum Numbers (BPNs), which serve as indicators of skid resistance, across various sections of the pavement panel specimens. Skid resistance measurements were conducted at the central zone of each scanned area. The testing was conducted under controlled environmental conditions (20 ± 1 °C ambient temperature, 60 ± 5% relative humidity) in full compliance with the operational specifications outlined in EN 13036-4 for pendulum measurements [27].

### 2.4. Linear Laser Selection

In the realm of linear laser scanning technology, red and blue laser modules are extensively utilized. This study assessed the suitability of these modules for scanning the texture of asphalt pavements by conducting experiments with both red and blue linear lasers on asphalt surfaces. The specific parameters of the lasers used are detailed in Table 2, playing a crucial role in the evaluation of scanning results and in identifying the optimal laser module for pavement texture scanning.

**Figure 6:**
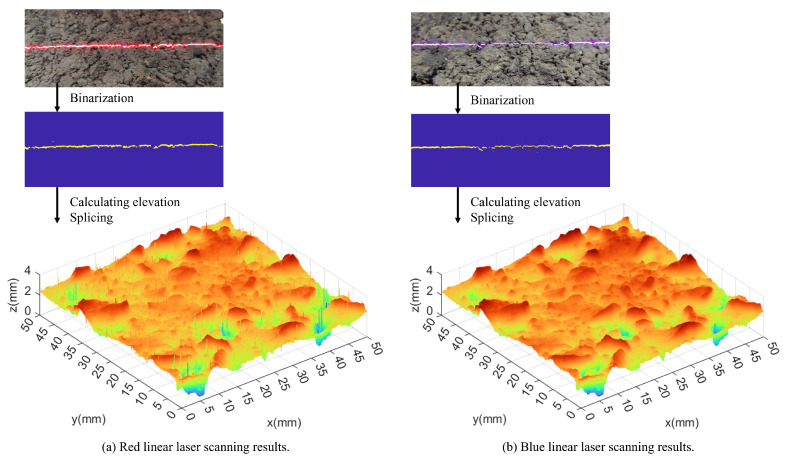
Comparison of asphalt pavement texture scanning results using red and blue linear lasers.

The experimental outcomes depicted in Figure 6 demonstrate the application of red and blue linear laser scanning technologies for assessing the texture of asphalt pavement surfaces. The analysis of the captured texture images reveals that the red laser, with its wider line width and greater tendency to scatter, results in a more extensive dispersion of point clouds in the scanned texture. Notably, the point cloud data from the red linear laser scanning shows a higher frequency of isolated points, potentially due to the laser’s scattering effect, which leads to a more diffuse reflection on the pavement surface. In contrast, the images obtained via blue linear laser scanning present a denser and more unified texture depiction, implying that the blue laser offers enhanced precision and resolution in texture scanning tasks. Therefore, in terms of point cloud image quality and the clarity of textural features, the blue linear laser is considered more suitable for scanning the texture of asphalt pavement. Consequently, the blue linear laser was chosen as the preferred laser source for texture extraction in subsequent analyses of asphalt pavement.

The parameters of the device are shown in Table 3.

### 2.5. Pre-Processing of Pavement Texture Data

The initially extracted point cloud file appeared reflective due to the presence of a small amount of oil on the surface of the asphalt, and there was a small amount of noise such as scattered points and isolated points. Moreover, the asphalt specimen may have been tilted due to the angle between the asphalt specimen and the scanning instrument during the scanning process [28]. To ensure result accuracy, the pavement texture data were pre-processed before data acquisition.

Road surface texture data pre-processing was divided into two steps; the first step was to remove noise from the original surface texture using a Gaussian filter. A Gaussian filter processes a data point and the surrounding n data points by the weighted average of the normal distribution probability density function. The probability density function is shown in Equation (6). Points that are much larger or smaller than the average value are identified as noise; after, the identified noise is calibrated for the removal of the noise. In this study, a Gaussian filter with a wavelength of 0.5 mm was used to remove noise.

(6) $$h\left(x,y\right)=\frac{1}{2\pi \sigma^{2}}e^{-\frac{x^{2}+y^{2}}{2\sigma^{2}}}$$

where (x, y) are the coordinates of the data point and h(x, y) is the elevation of that data point.

**Figure 7:**
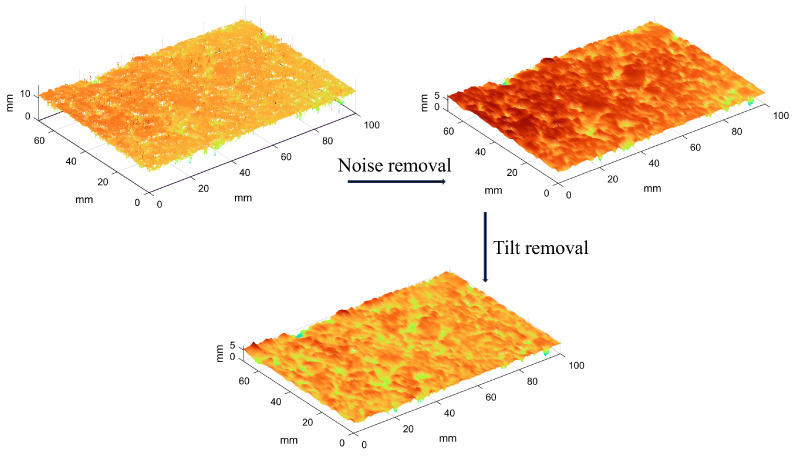
Schematic diagram of pavement data pre-processing.

The second step of data pre-processing was to level the asphalt pavement texture, which was based on the principle that the inclined pavement cross-section could be used with the least-squares method to fit a straight line to derive the straight line slope k; then, the inclined pavement cross-section in accordance with the slope of k for the rotation of the inclined pavement texture data could be leveled. The k-value formula is shown in Equation (7), and the pre-processing of the data is illustrated in Figure 7.

(7) $$k=\frac{\sum xy-n\bar{x}\bar{y}}{\sum x^{2}-n{\bar{x}}^{2}}$$

### 2.6. Fractal Dimension Calculation

Fractal analysis is frequently used to quantify the roughness and irregularity of fracture surfaces [29,30,31]. The current extraction of the fractal dimension of asphalt sections was performed using box-counting method.

The asphalt pavement was positioned within a uniformly partitioned square grid, and the count of the minimum boxes necessary to cover the pavement surface was determined. By employing various observational scales, the corresponding number of boxes was ascertained, essentially scaling the dimensionality. Here, the length of the box edges was denoted by σ, and the number of boxes required to cover the surface was represented by N(σ). Consequently, this relationship is encapsulated in Equation (8).

(8) $$N\left(\sigma \right)∼\sigma^{-D}$$

where D is the fractal dimension, from which N (σ) grows in a square relationship with 1/σ, leading to the calculation of the fractal dimension D [32] (Equation (9)).

(9) $$D=\lim_{\sigma \to 0}\frac{\ln N(\sigma )}{\ln\left(\frac{1}{\sigma }\right)}$$

**Figure 8:**
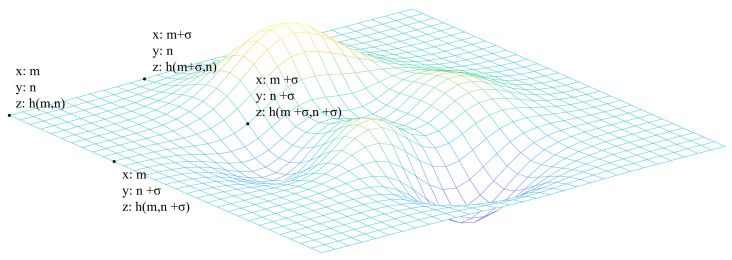
Schematic diagram of fractal dimension calculation.

In calculating the number of boxes covering the asphalt pavement surface, we utilize a grid algorithm, assuming that Sσ,m,n is the set of elevations of all the asphalt pavement texture collection points in the box with a box side length of σ in the direction of the plane of the xy-coordinate axis in the range bounded by the coordinate points of (m, n), (m + σ, n), (m, n + σ), and (m + σ, n + σ), as shown in Figure 8.

(10) $$S_{\sigma ,m,n}=\left\{h\left(m,n\right),\ldots ,h\left(m+\sigma ,n+\sigma \right)\right\}$$

Deriving hmax as the value of the elevation of the highest point in the set of Sσ,m,n and hmin as the value of the elevation of the lowest point in the set of Sσ,m,n, it is possible to derive the number of boxes used in the box with the side length σ, in the direction of the plane of the xy-coordinate axis, within the range bounded by the coordinate points (m, n), (m + σ,n), (m, n + σ), (m + σ, n + σ), and the number of boxes used in the box with the side length σ and in the direction of the plane of the xy-coordinate axis, enclosed with the coordinate points (m, n), (m, n + σ), and (m + σ, n + σ), is calculated by Equation (11).

(11) $$N_{\sigma ,m,n}=\frac{h_{\max}-h_{\min}}{\sigma }+1$$

Calculating the quantity of boxes for each coordinate range at an edge length of σ, we determine the total box count necessary to cover the textured surface by summing these quantities.

(12) $$N\left(\sigma \right)=\sum N_{\sigma ,m,n}$$

With constantly changing edge lengths (σ), the resulting box number N(σ) and the edge length σ are plotted as a scatter plot on a double logarithmic curve. A linear regression is then obtained for the scatterplot, and the slope of the resulting line can be approximated to be equal to the fractal dimension *D*, where the surface has the fractal dimension D∈[2,3).

The method was implemented using Matlab software, so the side length σ was 0.05 mm, 0.1 mm, 0.2 mm, 0.25 mm, 0.5 mm, 1 mm, 2 mm, 5 mm, and 10 mm, and the number of boxes N(σ) under different side lengths (σ) was calculated with the coordinates (0, 0) as the initial point, respectively, and, finally, the obtained N(σ) was plotted against the side lengths σ in a scatterplot on a double logarithmic curve. Then, linear regression was obtained for the scatter plot, and the slope of the obtained line was the fractal dimension D. The software used in this study is MATLAB, version 9.14.0.2206163 (R2023a).

The measured fractal dimension of a smooth plane, obtained via this technique, is 2.0000007, nearly identical to the expected value of 2.0. This proximity validates the method’s applicability for calculating surface fractal dimensions.

## 3. Results

**Figure 9:**
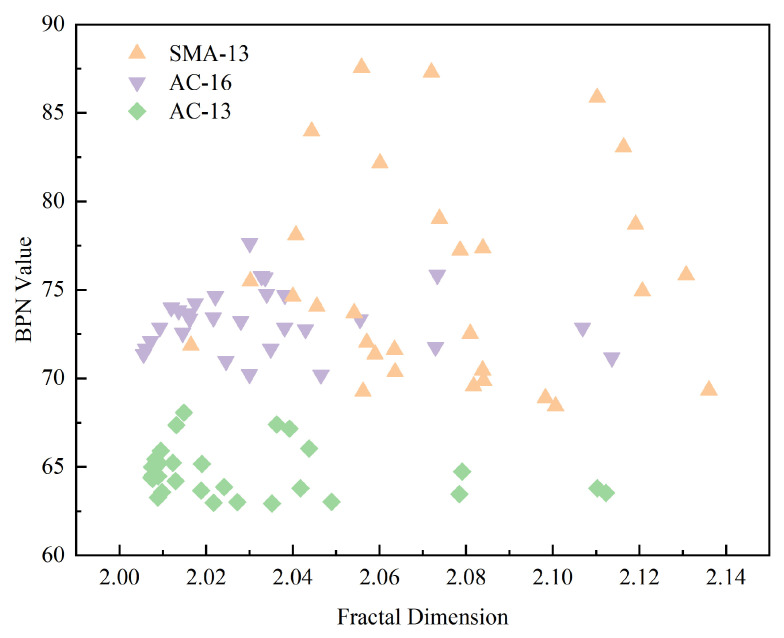
Relationship between BPN value and fractal dimension in full-depth asphalt pavement.

The fractal dimension of the overall asphalt pavement 3D texture cloud map was derived by the method described in Section 2.3, and Figure 9 illustrates the correlation between the fractal dimension of the full-depth asphalt pavement 3D cloud map and the BPN value.

From Figure 9, it can be found that the overall skid resistance is ranked as SMA-13 > AC-16 > AC-13. Significant disparities in skid resistance are observable among various asphalt pavement types, even when they possess comparable fractal dimensions. And, there is no significant regular increase in the BPN value as the fractal dimension increases. This indicates that there is no significant correlation between asphalt pavement skid resistance and the fractal dimension of the overall asphalt pavement 3D texture cloud map. The variation in skid resistance among different types of asphalt pavements, despite similar fractal dimensions, could be attributed to the limited area that contributes to skid resistance during the skid resistance test. Additionally, it might be due to the substantial differences in textural characteristics across various gradation types of asphalt pavements.

Therefore, different gradation types of asphalt pavement textures were classified, and the fractal dimensions of asphalt pavements at different depths were calculated and correlated with skid resistance to find out the EDSR.

### 3.1. Fractal Dimensions at Different Depths

**Figure 10:**
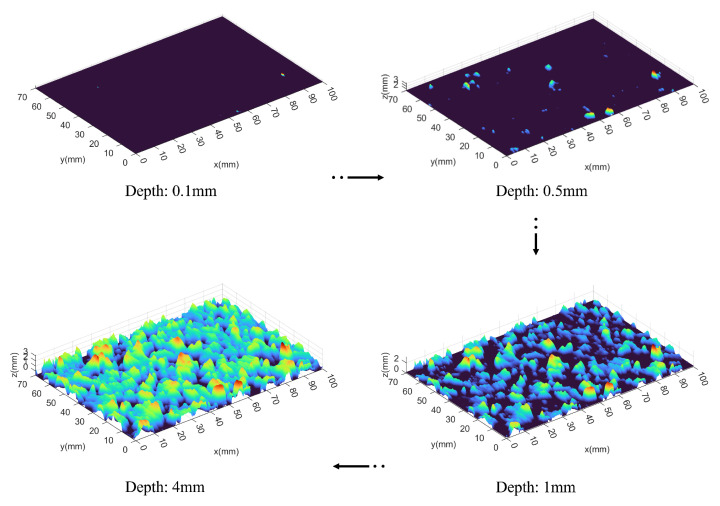
Three-dimensional cloud map of pavement at different depths.

After following the procedure in Section 2, we obtained the surface texture of nine specimens. To determine the fractal dimension of asphalt pavement at various depths, the procedure commenced with designating the plane at the apex of the 3D cloud map as the uppermost texture surface. Subsequently, the pavement’s 3D cloud maps were sequentially extracted, each at a depth interval of 0.1 mm, to analyze the textural features at different levels. This is schematically shown in Figure 10. In this work, we assigned the elevation of the point cloud below the selected range to the elevation of the lowest point to ensure the completeness of the plane and to facilitate the calculation of the fractal dimension.

After layering the 3D cloud map of asphalt pavement, as shown in Figure 10, the fractal dimensions of all the generated 3D cloud maps were obtained.

**Figure 11:**
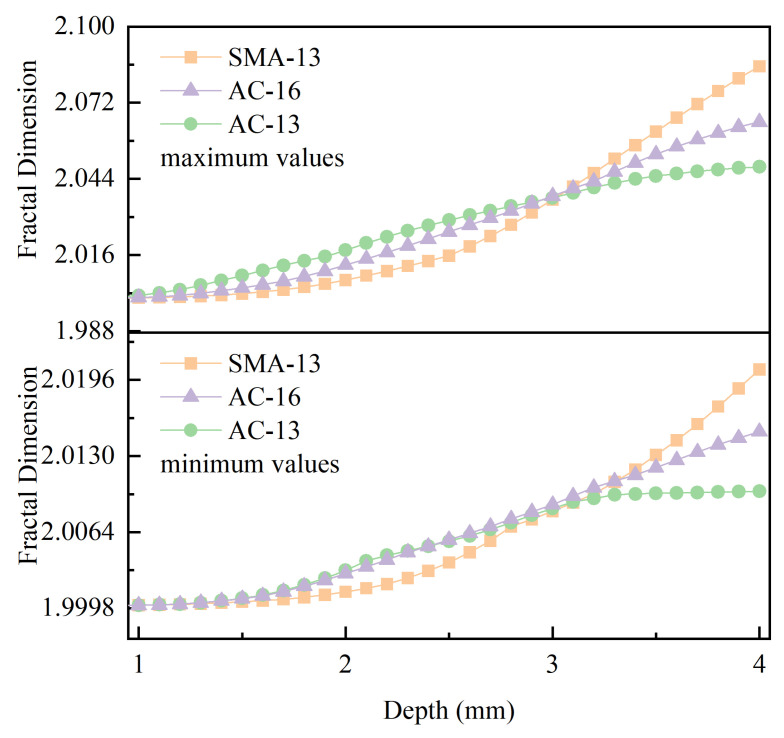
Fractal dimension envelope plot.

Figure 11 illustrates the envelope of the fractal dimension of all measured pavements of SMA-13, AC-13, and AC-16 as a function of depth. From Figure 11, it can be seen that at depths of 1–3 mm, the fractal dimension as a whole shows a trend of AC-13 > AC-16 > SMA-13, while at 3–4 mm, the results are completely opposite, showing a trend of SMA-13 > AC-16 > AC-13.

### 3.2. Pavement Texture Analysis

**Figure 12:**
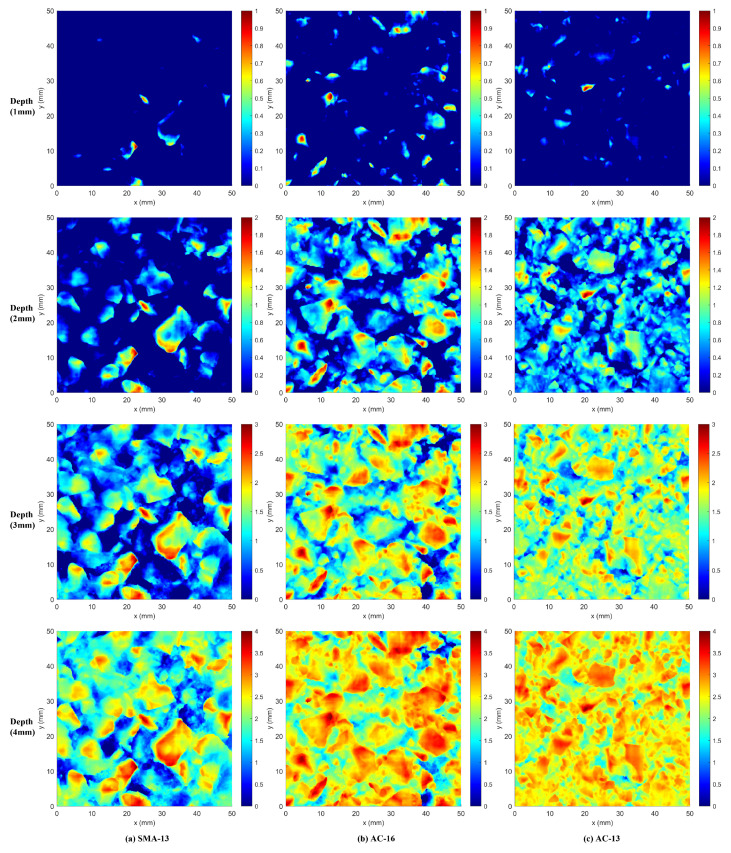
Elevation maps at different depths.

In order to more visually analyze the texture distribution of different types of asphalt pavements at different depths, Figure 12 demonstrates the elevation maps of SMA-13, AC-16, and AC-13 asphalt pavements at different depths for asphalt pavements at 1 mm, 2 mm, 3 mm, and 4 mm depths, respectively. The range was set to 50 mm × 50 mm for ease of observation and the lowest point of each floor elevation map was set to 0 mm.

From Figure 12, we can see that the asphalt pavement texture gradually enriches with increasing depth. The texture distribution of open-graded asphalt pavements (SMA-13) at depths of 1 mm and 2 mm is significantly lower than that of dense-graded asphalt pavements (AC-13, AC-16) due to the larger voids in the pavement; at the depths of 3 mm and 4 mm, the open-graded asphalt pavement (SMA-13) point cloud elevation is mainly distributed in the lower part of the depth, while the intensively graded asphalt pavement (AC-13, AC-16) point cloud is mainly distributed in the upper part of the depth.

This also illustrates that the fractal dimension of the texture of the open-graded asphalt pavement (SMA-13) at depths of 1–3 mm in Figure 11 is smaller than that of the dense-graded asphalt pavements (AC-13, AC-16) due to its lower texture distribution than that of the dense-graded asphalt pavements (AC-13, AC-16).

### 3.3. Abnormal Data Removal

**Figure 13:**
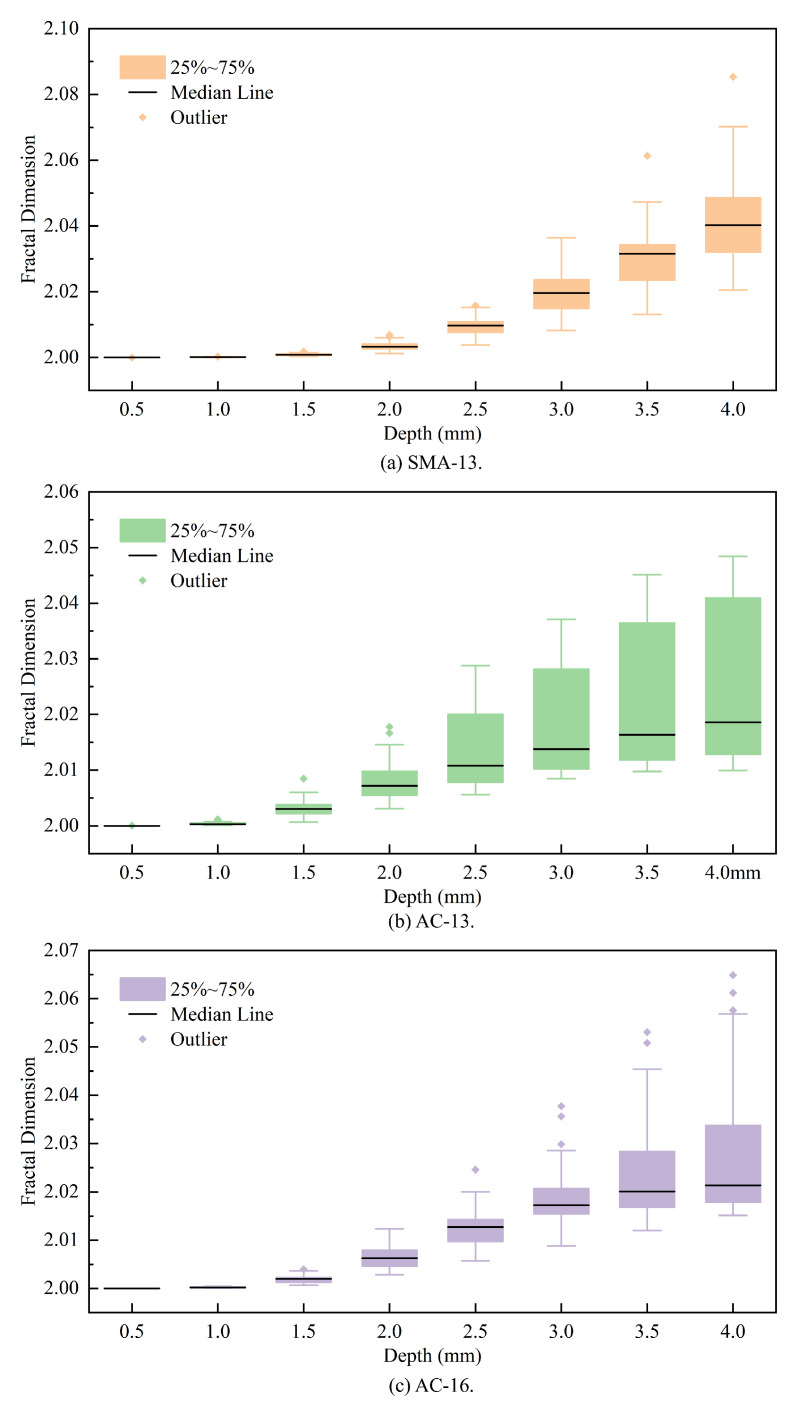
Box plots of fractal dimensions.

To analyze the distribution of the differences in the asphalt pavement at different depths, we selected the depth of 0.5 mm as the interval of the asphalt pavement fractal dimensions to construct box plots, as shown in Figure 13.

Figure 13 indicates that the minimum, maximum, and median values of the fractal dimensions for the three asphalt pavement types increase as depth progresses. In both SMA-13 and AC-16, anomalies much larger than the average fractal dimension appear with increasing depth; in AC-13, the opposite is true, with the anomalies disappearing with increasing depth.

The fractal dimension distribution of all three types of asphalt pavements exhibits a progressive broadening with increasing depth, signifying an escalating disparity in the fractal dimensions within the same pavement category. At a depth of 4 mm, the range of variation in the fractal dimensions for these pavements is maximized, with the differences reaching 0.09 for SMA-13, 0.082 for AC-16, and 0.088 for AC-13, respectively.

**Figure 14:**
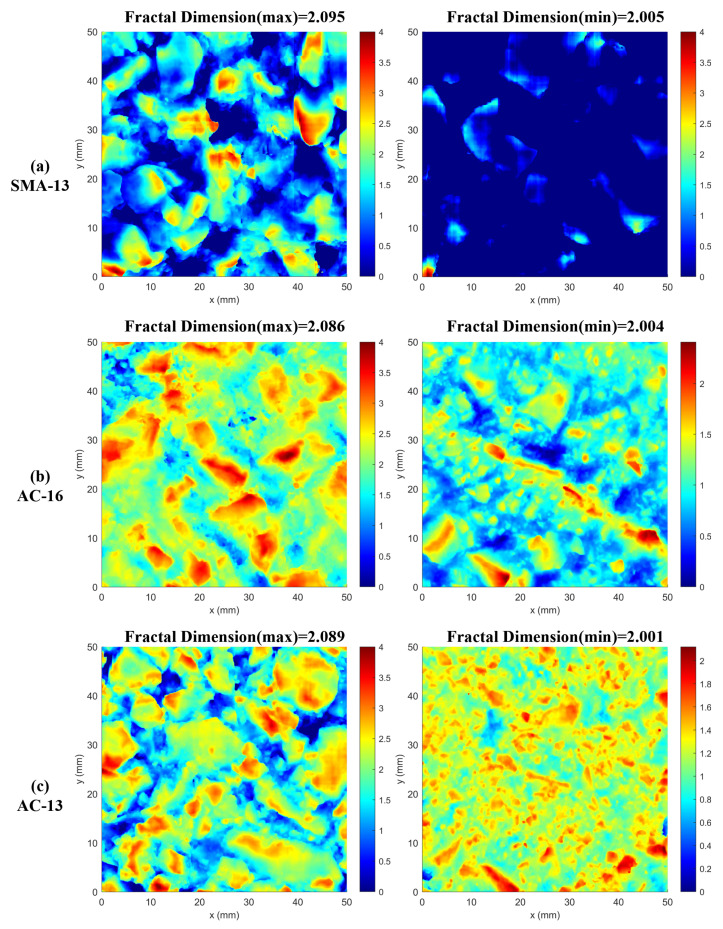
Elevation maps at maximum and minimum fractal dimensions.

In order to visually analyze this discrepancy and the occurrence of outliers, the elevation maps corresponding to the maximum and minimum fractal dimension values of the three types of asphalt pavements at a depth of 4 mm were selected, as shown in Figure 14.

From Figure 14, we see that the fractal dimension of the SMA-13 type of asphalt pavement is the largest when its pavement elevation distribution is uniform; the fractal dimension is the smallest when there is a high protruding stone on the pavement resulting in a large deviation in the texture distribution of the asphalt pavement, and, therefore, its fractal dimension is lower.

In the case of the AC-16 and AC-13 asphalt pavements, the distribution of pavement elevation is observed to be uniform when the fractal dimension attains its maximum value. Conversely, when the fractal dimension is at its minimum, the elevation of both pavement types is found to be less than 4 mm. This phenomenon can be attributed to the wear and tear of the pavement surface over time, primarily due to wheel abrasion and other factors. Such wear is most pronounced at depths less than 3 mm, which in turn results in a reduced fractal dimension.

To ensure the accuracy of subsequent data analyses, we excluded outliers from the SMA-13 pavement data, which were attributed to individual aggregate bulges. In response to the limited depth data in the AC-13 and AC-16 pavements, we omitted areas with depths below 3 mm. Given that the majority of the pavement depths were generally less than 4 mm, we adjusted the depth range for these pavements to 0 mm to 3 mm for all subsequent analyses.

### 3.4. Correlation Analysis Between Textural Fractal Dimension and Pavement Friction

**Figure 15:**
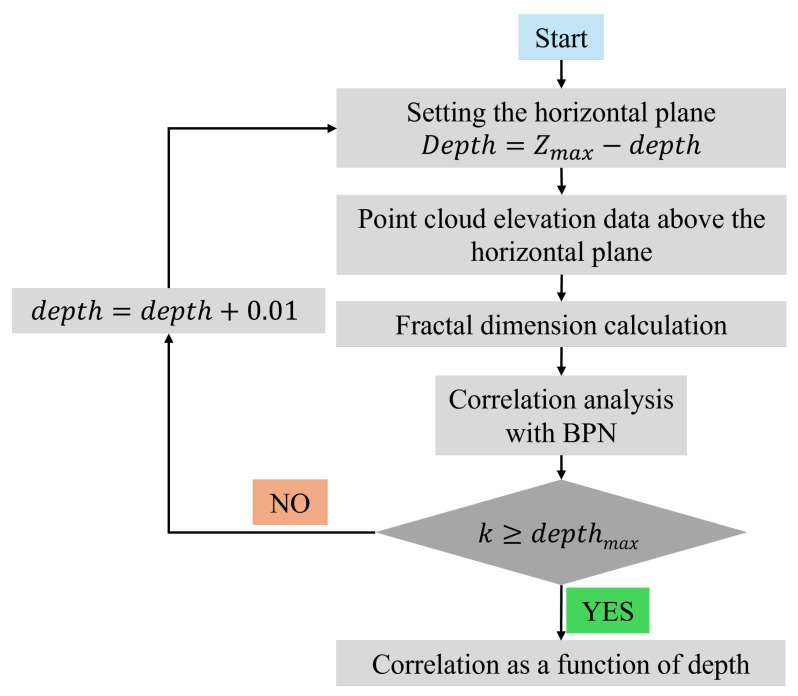
Textural fractal dimension and pavement friction correlation analysis flowchart.

To elucidate the relationship between the fractal dimension of the asphalt pavement and its frictional properties, we correlated the BPN values, obtained from the experimental measurements of the asphalt mixtures, with the fractal dimensions. The correlation between these two variables is typically quantified using the correlation coefficient R-value, as detailed in Equation (13).

(13) $$r=\frac{\sum_{i=1}^{n}\left(X_{i}-\bar{X}\right)\left(Y_{i}-\bar{Y}\right)}{\sqrt{\sum_{i=1}^{n}{\left(X_{i}-\bar{X}\right)}^{2}}\sqrt{\sum_{i=1}^{n}{\left(Y_{i}-\bar{Y}\right)}^{2}}}$$

where Xi is the value of the fractal dimension of the 3D cloud map of the type i asphalt mixture at different depths. $\bar{X}$ represents the mean fractal dimension of the 3D texture across various depths. Yi denotes a specific British Pendulum Number (BPN) for the type i asphalt mixture. $\bar{Y}$ is the overall average BPN value for the entire set of asphalt mixtures. The calculation flowchart is shown in Figure 15.

**Figure 16:**
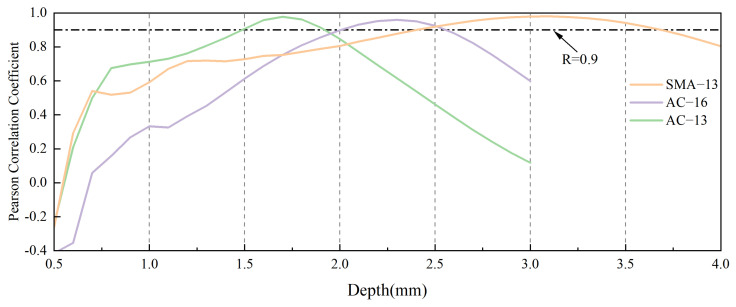
Plot of correlation coefficients between BPN values and fractal dimensions at different depths.

Figure 16 shows the correlation between the fractal dimensions from 3D point cloud maps and the measured BPN values for AC-13, AC-16, and SMA-13 asphalt pavements at various depths. A correlation coefficient (R-value) exceeding 0.9 is conventionally indicative of a strong correlation. We define this section as the EDSR. As depicted in Figure 16, the EDSR at which this is observed varies among different grades of asphalt pavements. For AC-13, the EDSR range is 1.5 mm to 1.8 mm; for AC-16, it is within 2.0 mm to 2.6 mm; and for SMA-13, the range is 2.4 mm to 3.6 mm.

**Figure 17:**
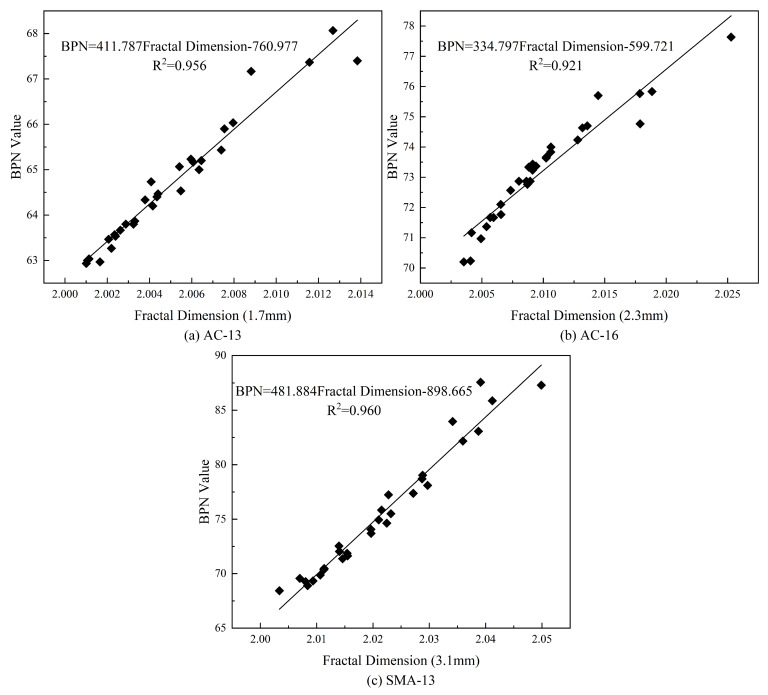
Plots of best-fit BPN values versus fractal dimension fits.

Figure 17 illustrates the linear regression fit for the relationship between the fractal dimension value and the BPN value for the three types of asphalt pavements, showcasing the highest degree of fit and providing the corresponding linear equation. Figure 16 reveals that the coefficients of determination (R^2^) for AC-13, AC-16, and SMA-13 at the depth that contributes most effectively to skid resistance are 0.956, 0.921, and 0.960, respectively. These high R^2^ values suggest that the fractal dimension at this optimal depth can serve as a reliable indicator of the skid resistance for each type of asphalt pavement.

### 3.5. Validation of Skid Resistance Model

To further validate the applicability of the skid resistance calculation model proposed in Figure 17, this study followed the operational steps detailed in Section 2, selecting nine sample areas on three different types of asphalt pavements to calculate skid resistance.

**Figure 18:**
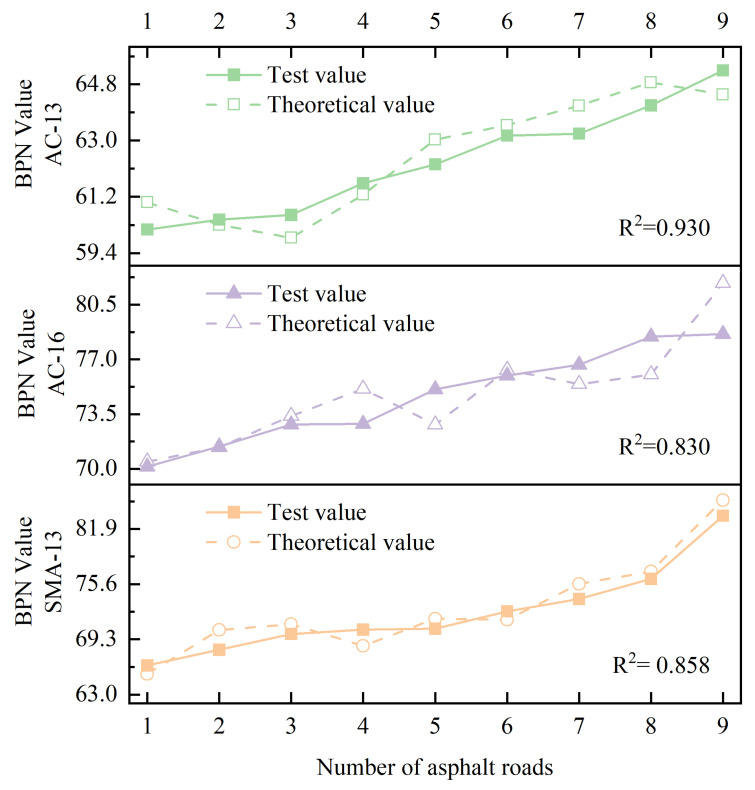
Comparison of predicted and actual skid resistance for different asphalt pavements.

Subsequently, a comprehensive comparison analysis was conducted between the calculated results and the actual measured data, with the related comparison results detailed in Figure 18. According to the data shown in Figure 18, it can be clearly observed that the model proposed in this study had high accuracy in detecting actual skid resistance, with the coefficient of determination (R^2^) exceeding 0.83 in all cases. This statistical indicator signifies that the model can reliably simulate the skid resistance of asphalt pavements.

## 4. Conclusions

In this research, the surface textures of three asphalt pavements, AC-13, AC-16, and SMA-13, were meticulously captured using a high-resolution 3D laser scanner. Concurrently, the surface friction coefficients were precisely measured by employing a British pendulum. The fractal dimensions of the pavement surface textures at various depths were determined through the application of the three-dimensional box-counting method. Subsequently, these fractal dimensions were correlated with the coefficient of kinetic friction to perform a comprehensive analysis. The findings of this investigation are encapsulated in the following conclusions:

1. The research found that the fractal dimension of asphalt pavement serves as an effective indicator of the pavement’s overall roughness. However, no significant correlation was observed between the fractal dimension and the pavement’s skid resistance performance. Considering this finding, the concept of the “EDSR (Effective Depth of Skid Resistance)” was introduced to better understand and quantify the depth at which pavement surface characteristics contribute most effectively to skid resistance.
2. Through an analytical approach that assessed the relationship between pavement fractal dimensions at different depths and the BPN values, this research pinpointed the EDSR ranges for various asphalt wearing courses with different aggregate grain sizes. For dense-graded AC-13, the interval of pronounced correlation is confined between 1.5 mm and 1.8 mm, while for AC-16, it spans from 2.0 mm to 2.6 mm. Open-graded SMA-13 reveals a strong correlation within a deeper range of 2.4 mm to 3.6 mm.
3. The linear regression analysis of fractal dimensions and BPN values for AC-13, AC-16, and SMA-13 asphalt pavements, conducted at the depths that contribute most to skid resistance, yielded R^2^ values of 0.956, 0.921, and 0.960, respectively.
4. This study aimed to create a reliable theoretical model for predicting asphalt pavement skid resistance. Validated against real-world data, our model demonstrated over 80% accuracy in all instances.

## Figures and Tables

**Table 1:** Asphalt mixture gradation.

Asphalt Mixture	Pass Rate Through Different Apertures (mm)/%										
	19	16	13.2	9.5	4.75	2.36	1.18	0.6	0.3	0.15	0.075
AC-13	100	100	95	77	53	37.5	27.5	18.5	14	11	6.5
AC-16	100	95	83	67	46	31	24.5	17.5	12.5	9.5	6
SMA-13	100	100	96	65	27	18	20	15	11.5	9.5	8.5

**Table 2:** Laser parameters.

Parameter Name	Blue Laser Parameter Values	Red Laser Parameter Values
Wavelength	405–500 nm	630–750 nm
Power	70 mw	30 mw
Line width	100–300 μm	650 nm
Focal length	130 mm	130 mm
Depth of field	100 mm	100 mm
Linearity	<0.1%	<0.8%
Uniformity	>85%	>75%

**Table 3:** Laser texture scanner parameter table.

Parameter Name	Parameter Value
Vertical resolution	0.003 mm
Measured mileage (depth)	30 mm
Maximum vertical resolution	0.00635 mm
Maximum horizontal resolution	0.0247 mm
Single scanning area	104 mm × 72 mm
Maximum sampling speed	3 kHz
Output file type	point cloud file
Data storage	via network cable
Instrument size	800 mm × 685 mm × 610 mm
Weights	12.5 kg

## Data Availability

The original contributions presented in this study are included in the article. Further inquiries can be directed to the corresponding authors.
